# Factors Associated With the Use of Strong Opioids Among First-Time Patients at a Colombian Pain Clinic: A Cross-Sectional Study

**DOI:** 10.7759/cureus.95406

**Published:** 2025-10-25

**Authors:** Juan Carlos Amaya-Rios, Adriana Marrugo-Gomez, Andrea García

**Affiliations:** 1 Pain Medicine, Clínica Infantil Santa María del Lago, Bogotá, COL; 2 Pain Medicine, Clinica Infantil Santa Maria del Lago, Bogotá, COL

**Keywords:** opioid, opioid epidemic, opioid-related disorders, pain clinics, pain management, prescriptions

## Abstract

Chronic pain continues to have an important burden on disability-adjusted life years, and its adequate management remains a challenge for medical personnel. Opioids have been used throughout history to treat pain and are considered the most effective pain medications; however, they come with their own undesirable effects, such as addiction, which has created a worldwide opioid crisis. Our aim was to identify factors associated with strong opioid use in patients consulting for the first time at a pain clinic in Bogotá, Colombia.

An observational transversal cohort study was conducted to determine the prevalence and associated factors of strong opioid use in non-oncologic patients attending for the first time a pain clinic outpatient consult in Bogotá, Colombia. Two hundred one patient records were included for evaluation, and an 18% prevalence was found. Bivariate and multivariate regression analyses were performed to find factors associated with strong opioid use.

Being male (OR: 1.57) and having psychiatric disease (OR: 1.5) were risk factors for the prescription of strong opioids; however, no statistical significance was found with any of the studied factors.

Further studies are needed to completely identify the associated factors of opioid prescription and use in non-oncologic patients before their attendance at a pain clinic, and this finding will be useful to improve pain management education among non-pain specialists.

## Introduction

Chronic pain continues to represent a significant burden on disability-adjusted life years; eight of the 12 main non-transmissible disabling diseases are painful conditions or psychiatric disorders associated with pain. It accounts for approximately 15% of physician visits and has a prevalence of around 20% worldwide [1,2], reaching up to 26% in Colombia. Pain management associations, as well as the World Health Organization (WHO), have launched multiple strategies to help physicians deliver safer and more effective pain relief treatments, the most well-known being the analgesic ladder introduced by the WHO for cancer pain relief [3]. This strategy has been updated since its first release. However, its application to patients with chronic non-cancer pain has been debated, especially as the importance of non-pharmacologic, multidisciplinary, and multimodal management has been increasingly acknowledged. This has led to the addition of new steps to the ladder and the development of alternative strategies [4].

Opioids have been used for pain relief for thousands of years, with reports of their use dating back to ancient Egypt. Today, they remain the most potent analgesics [5,6]. They are mu-receptor agonists that activate the endogenous opioid system, which is involved in various bodily functions beyond pain modulation, making side effects almost unavoidable [7]. These side effects depend on opioid potency, dosage, and duration of exposure [7]. Prolonged exposure is associated with a loss of mu receptors, which may contribute to increased pain sensitivity and opioid-induced hyperalgesia [8]. Evidence supports short-term opioid use for reducing pain and improving functionality in non-oncologic pain; however, these benefits diminish beyond 12 weeks of use [9].

Pain management strategies commonly categorize opioids into weak and strong classes based on an arbitrary differentiation of their potency, using them to treat either mild-to-moderate or moderate-to-severe pain, respectively [3]. Potency is influenced by receptor affinity and efficacy. Thus, sufentanil, fentanyl, buprenorphine, morphine, hydromorphone, oxycodone, and methadone are considered strong opioids, while codeine, hydrocodone, tramadol, and tapentadol are categorized as weak [10]. However, differences in mechanisms of action and individual patient variations, especially cytochrome system polymorphisms, play a crucial role in the analgesic effect [10]. In Colombia, weak opioids are often sold over the counter in combination with acetaminophen, leading to the misconception that they are less effective, even though some are equianalgesic to morphine [11]. This availability remains common practice despite opioids requiring a prescription for access.

Multiple factors have contributed to the global opioid crisis, including the aforementioned ease of access and a general sense among medical personnel of being undertrained in chronic pain management, leading to concerns about inappropriate usage [12]. Over the past two decades, there has been a worldwide increase in opioid prescriptions. In 2016, there were 63,632 overdose-related deaths in the United States, 66.4% of which were associated with opioids. This led the CDC to publish guidelines for opioid prescription in chronic pain, aiming to reduce risks associated with prolonged use, such as misuse, overdose, and death [9,13].

In Colombia, studies have identified differences in analgesic prescription patterns, with higher opioid prescription rates in capital cities compared with rural areas [14]. However, to the best of our knowledge, no studies have assessed other factors associated with opioid prescriptions, particularly strong opioids, in Colombia. Therefore, this study aims to identify factors related to strong opioid prescription among patients consulting for the first time at a pain clinic in Bogotá, Colombia.

## Materials and methods

An observational, cross-sectional cohort study was conducted to determine the prevalence of and factors associated with strong opioid use in non-oncologic patients attending a pain clinic outpatient consultation for the first time in Bogotá, Colombia. Patients included were first-time consultants between September 2021 and September 2022, aged over 18 years, with a chronic pain diagnosis (defined as pain lasting more than three months), and an opioid prescription. Patients with an active cancer diagnosis or incomplete records were excluded.

The study protocol was approved by the Ethics and Research Committee at Fundación Universitaria Sanitas. Upon approval, the need for informed consent was waived. Patient record data included demographics, pain intensity (multiple pain evaluation instruments were reported in patient records; all pain intensity evaluations were converted to a four-point categorical scale: 0 = no pain, 1-3 = mild, 4-6 = moderate, and >7 = severe), pain-related diagnosis, opioid prescriptions, prescriber, and the conduct of the pain specialist.

A descriptive analysis was performed comparing characteristics of patients using strong opioids with those who were not. The chi-square test or Fisher’s exact test was used for qualitative variables, and either Student’s t-test or non-parametric tests were used for quantitative variables, depending on distribution. After calculating the prevalence of strong opioid use, a bivariate regression was run for independent variables, followed by a multivariate regression using clinically relevant variables with biological plausibility to identify associated factors. Sample size was calculated using Freeman’s formula: n = 10 × (k + 1), with five explanatory variables included for the multivariate analysis and a 33% prevalence, yielding a sample size (n) of 200 patients [15,16]. A p-value <0.05 was considered statistically significant. All analyses were performed using R version 4.0.3 (R Foundation for Statistical Computing, Vienna, Austria).

## Results

After assessing 1,200 patient records and applying inclusion and exclusion criteria, 201 patients were selected.

Patient characteristics

The median age was 61 years, and 149 patients were women. Pain intensity was reported as severe by 113 patients, moderate by 85, and mild by only three. Pain duration was reported as 3 months to 1 year in 20%, 1-5 years in 57.7%, and longer than 5 years in 20.9%. The most frequent diagnosis was lumbar pain (59.2%), followed by arthrosis (27.9%), shoulder pain (11.9%), fibromyalgia (11.4%), and neck pain (10.9%). Other diagnoses included rheumatologic disorders, fractures, headaches, and amputations. Psychiatric comorbidity was present in 18% of patients, with anxiety and depression being the most common. Table 1 presents the patient characteristics.

**Table 1:** Clinical characteristics of study participants according to strong opioid use Q1 = Quartile 1; Q3 = Quartile 3. *Psychoactive substance use, somatoform disorder, opioid dependence, obsessive-compulsive disorder. ^**^Student t test = 0.7518. ^***^Chi^2 ^= 1.5649. ​​​​​​​^****^U-Mann Whitney. ​​​​​​​^*****^Fisher. ​​​​​​​^******^Chi^2 ^= 2.2187. ​​​​​​​^*******^Chi^2 ^= 0.7891.

Variable	All	Strong opioid		P
	(n = 201)	No (n = 166)	Yes (n = 35)	
Age, median (±)	60.8 (15.9)	61.17 (15.81)	58.94 (16.42)	0.453**
Sex, n (%)				0.211***
Female	149 (74.1)	126 (75.9)	23 (65.7)	
Male	52 (25.9)	40 (24.1)	12 (34.3)	
Pain intensity, median (Q1-Q3)	8 (6-8)	8 (7-8)	7 (6-8)	0.362^****^
Pain level, n (%)				0.345^*****^
Mild	3 (1.5)	2 (1.2)	1 (2.8)	
Moderate	85 (42.3)	68 (41.0)	17 (48.6)	
Severe	113 (56.2)	96 (57.8)	17 (48.6)	
Pain duration, n (%)				0.330******
Less than a year	40 (19.9)	30 (18.1)	10 (28.6)	
1-5 years	116 (57.7)	99 (59.6)	17 (48.6)	
Over 5 years	45 (22.4)	37 (22.3)	8 (22.8)	
Diagnosis, n (%)				0.444^*****^
Low back pain	119 (59.2)	97 (58.4)	22 (62.9)	
Arthrosis	56 (27.9)	47 (28.3)	9 (25.7)	
Shoulder pain	24 (11.9)	22 (13.3)	2 (5.7)	
Fibromyalgia	23 (11.4)	20 (12.0)	3 (8.6)	
Neck pain	22 (10.9)	20 (12.0)	2 (5.7)	
Rheumatologic condition	18 (9.0)	12 (7.2)	6 (17.1)	
Fracture	11 (5.5)	6 (3.6)	5 (14.3)	
Headache	2 (1.0)	1 (0.6)	1 (2.8)	
Amputation	2 (1.0)	2 (1.2)	0 (0.0)	
Other	32 (15.9)	27 (16.3)	5 (14.3)	
Comorbidities, n (%)				1.000^*****^
Hypertension	75 (37.3)	61 (36.6)	14 (40.0)	
Diabetes mellitus	31 (15.4)	23 (13.9)	8 (22.9)	
Peripheral venous insufficiency	4 (2.0)	4 (2.4)	0 (0.0)	
Stroke sequelae	3 (1.5)	2 (1.2)	1 (2.8)	
Chronic obstructive pulmonary disease	9 (4.5)	6 (3.6)	3 (8.6)	
Osteoporosis	23 (11.4)	17 (10.2)	6 (17.1)	
Hypothyroidism	36 (17.9)	32 (19.3)	4 (11.4)	
Chronic renal disease	16 (8.0)	12 (7.2)	4 (11.4)	
Other	84 (41.8)	73 (44.0)	11 (31.4)	
None	50 (24.9)	41 (24.7)	9 (25.7)	
Psychiatric diagnosis, n (%)	36 (17.9)	28 (16.9)	8 (22.9)	0.374*******
Anxiety	13 (36.1)	9 (32.1)	4 (50.0)	
Depression	9 (25.0)	6 (21.4)	3 (37.5)	
Mixed anxiety and depression disorder	9 (25.0)	6 (21.4)	3 (37.5)	
Adjustment disorder	4 (11.1)	3 (10.77)	1 (12.5)	
Bipolar disorder	3 (8.3)	2 (7.1)	1 (12.5)	
Other*	4 (11.1)	3 (10.77)	1 (12.5)	

Opioid prescription

The prevalence of strong opioid use was 18.4% (n = 37), with hydromorphone being the most common (7.5%), followed by oxycodone (6%) and buprenorphine (1.5%). Two patients had prescriptions for two strong opioids: hydromorphone with buprenorphine and hydromorphone with oxycodone. Among those using weak opioids, tramadol accounted for 35.3% (n = 71), hydrocodone for 24.4% (n = 49), and codeine for 17.9% (n = 36).

Non-opioid analgesic prescription by non-pain specialists

Concomitant analgesic use included acetaminophen (51.2%, n = 103), gabapentinoids (25.9%, n = 52), and non-steroidal anti-inflammatory drugs (NSAIDs) (20%, n = 39). Other antineuropathic medications, such as tricyclic antidepressants and duloxetine, were used less frequently.

Opioid misuse

Six patients were diagnosed with opioid misuse: two related to tramadol (22.5 and 70 morphine milligram equivalents per day (MME/D)), one to oxycodone (40 MME/D), one to buprenorphine (60 MME/D), one to hydromorphone (75 MME/D), and one to both oxycodone and hydromorphone (110 MME/D).

Prescriber specialty

In most cases (44.3%), prescriber specialty was not recorded. Where available, general practitioners accounted for 17.9% of prescriptions, followed by orthopedic surgeons (11.4%).

Pain specialist conduct

Pain specialists repeated the existing opioid prescription in 24.9% of cases. For strong opioids, this conduct was observed in only 13% of cases. Rotation or discontinuation occurred in 76.5% of strong opioid users, as detailed in Table 2. Table 3 shows pain specialist conduct in relation to the use of concomitant analgesics and referrals. 

**Table 2:** Pain specialist conduct regarding opioids

Conduct	Strong opioid, n (%)	All opioids, n (%)
Rotation	19 (50)	59 (29.3)
Repeat prescription	5 (13)	50 (24.9)
Dosage adjustment	4 (10.5)	48 (23.9)
Discontinuation	10 (26.5)	44 (21.9)

**Table 3:** Other conducts by pain specialist NSAID: non-steroidal anti-inflammatory drug.

Variables	n (%)
Other analgesics prescribed	
Acetaminophen	136 (67.7)
Gabapentinoid	109 (54.2)
Duloxetine	20 (10.0)
Oral NSAID	18 (9.0)
Amitriptyline	16 (8.0)
Imipramine	8 (4.0)
Topical NSAID	7 (3.5)
Lidocaine patch	2 (1.0)
Lidocaine gel	1 (0.5)
Other	24 (11.9)
Referral to other specialties	
Psychology	2 (1.0)
Psychiatry	7 (3.5)
Toxicology	5 (2.5)
Alternative medicine	10 (5.0)
None	135 (67.2)
Other	52 (25.9)

Bivariate and multivariate regression analysis

A bivariate and multivariate logistic regression analysis was performed with clinically relevant variables, including sex, age, pain duration, psychiatric disease, and pain intensity. Being male (OR: 1.57; p = 0.281) and having a psychiatric disease (OR: 1.5; p = 0.395) emerged as potential risk factors for strong opioid prescription; however, no statistically significant associations were found. Multivariate regression results are presented in Table 4. 

**Table 4:** Multivariate logistic regression model for the use of strong opioids

Variable	OR	CI	p
Age	0.99	0.97-1.02	0.931
Masculine	1.57	0.69-3.55	0.281
Pain lasting longer than a year	0.55	0.23-1.32	0.182
Having a psychiatric diagnosis	1.50	0.59-3.75	0.395
Pain intensity	0.91	0.74-1.13	0.412

## Discussion

The most prescribed opioid by non-pain specialists was tramadol. This finding is consistent with previous studies conducted in France and Colombia, where tramadol has been reported among the top three most prescribed opioids [14,17]. We believe this may be partially due to the misconception that tramadol has a safer profile because it is classified as a weak opioid and is available “over the counter,” despite Colombian regulations. Notably, two patients diagnosed with opioid misuse were taking tramadol, which supports this assumption and highlights the need to address misconceptions regarding the safety of weak opioids. Although no statistical analysis was performed regarding this specific relationship, since it was beyond the scope of this study, it remains a noteworthy observation warranting further research, particularly in pursuit of stronger regulation of these medications [18,19].

The prevalence of strong opioid use during first-time consultations at our pain clinic was 18.4%, with hydromorphone and oxycodone being the most frequently prescribed. It is concerning that two patients were prescribed more than one strong opioid, a practice that could increase the risk of complications, especially in patients with non-oncologic pain. The use of adjuvants, such as gabapentinoids (25.9%), was also common. In 2023, Chan et al. conducted a longitudinal study across 65 countries analyzing prescription trends for gabapentinoids between 2008 and 2018 and found a steady increase. In Colombia specifically, there was an average annual increase of 10.48% in their use [20].

Multivariate analysis revealed four variables that modified the risk of strong opioid prescription. Male gender and the presence of a psychiatric diagnosis were associated with an increased risk, while having pain for more than one year and experiencing severe pain were associated with a decreased risk. However, none of these associations reached statistical significance, possibly due to our sample size. These findings align with those of Yucuman et al., who conducted an observational study analyzing 12,577 cases in Colombia with reported behavioral disorders related to opioid use. They found a male-to-female ratio of 2.9:1 [21].

To our knowledge, no prior studies have evaluated the clinical decisions made by pain specialists during initial consultations with patients already receiving strong opioids. In our study, pain specialists modified the existing opioid regimen, through dose adjustment, rotation, or discontinuation, in 75.1% of cases. In contrast, only 24.9% of prescriptions were maintained unchanged. Specifically, for strong opioids, continuation occurred in just 13% of cases, while discontinuation occurred in 26.5%. Even without information on the decision-making process or reasons for discontinuation or dosage adjustment by the pain specialist, as they are beyond our scope, these findings suggest that pain specialists often disagree with previously prescribed analgesic regimens. This underscores the potential value of further research into how specialist training influences opioid prescribing behavior and how such training might contribute to addressing the opioid crisis. The observed differences in the concomitant use of other analgesics by pain specialists compared with other providers were notable, although a detailed analysis was beyond the scope of this study.

Among both strong and weak opioid prescribers, the most frequent specialties were general practitioners, orthopedic surgeons, internal medicine physicians, and neurosurgeons. Guy et al., in 2018, reported on opioid prescribing trends by specialty in the United States between 2016 and 2017. They found that the highest number of prescriptions came from internal medicine (16.4%), dentistry (15.8%), nurse practitioners (12.3%), and family medicine (10.3%). When grouped by care type, primary care accounted for 37.1% of prescriptions, followed by non-medical prescribers (19.2%) and pain specialists (8.9%). Other studies have ranked orthopedic surgeons higher, attributing such differences to variations in education and training among healthcare providers. These studies concluded that opioid prescribing guidelines should be tailored to these specific groups [22].

Even though 44.3% of our patient records lacked information on the prescribing specialist, and no statistically significant relationship was found between specialist type and strong opioid prescription, our findings are consistent with global trends. This suggests that efforts to improve prescribing behavior should focus on the same groups of medical professionals.

Inspired by studies conducted by Leon et al., which found that 83.7% of medical students had not received any specific coursework on pain management, 78% felt unprepared to manage oncologic pain, 44% were unsure about managing postoperative pain, and 73% lacked adequate knowledge of opioids, Gonima conducted a broad, non-structured review of pain management education in Colombia in 2019. He found that only 480 students had completed a pain and palliative care course during their medical education, representing just 9% of medical graduates in the country [23].

Simple interventions, such as providing feedback via email that compares surgical specialists’ prescribing patterns with those of their peers or with guideline standards, have been shown to improve prescription practices and reduce opioid use in the postoperative period [24]. This suggests that medical professionals are open to education and may be willing to change their prescribing behavior.

This study has limitations. As a cross-sectional, single-center study, it cannot establish causal relationships between the observed factors and strong opioid prescriptions. Therefore, larger multicenter and longitudinal studies should be conducted to comprehensively identify contributing factors. In addition, the relationship between pain improvement and specialist conduct regarding opioid treatment was not evaluated; we believe this aspect should be further explored in future research.

## Conclusions

Several factors may be associated with the prescription of strong opioids, including male gender, psychiatric comorbidities, and the medical specialty of the prescribing practitioner in the context of non-oncologic chronic pain. These findings are highlighted by the differences in the clinical approaches taken by pain specialists, not only regarding opioid prescriptions but also in their use of non-opioid analgesics and inter-specialty referrals, which are areas that warrant further investigation.

Our results demonstrate clear disparities in opioid prescribing practices across medical specialties for patients with non-oncologic pain. Therefore, institutional and national practice guidelines should prioritize targeted interventions aimed at specific medical settings and specialties. Efforts should begin in medical schools and residency programs by integrating comprehensive pain management education to ensure that future prescribers are equipped to manage chronic pain effectively. Combined with simple, evidence-based strategies, such efforts could help reduce inappropriate opioid prescriptions and address the opioid crisis more effectively.
